# Conjoined Use of EM and NMR in RNA Structure Refinement

**DOI:** 10.1371/journal.pone.0120445

**Published:** 2015-03-23

**Authors:** Zhou Gong, Charles D. Schwieters, Chun Tang

**Affiliations:** 1 CAS Key Laboratory of Magnetic Resonance in Biological Systems, National Magnetic Resonance Center at Wuhan, State Key Laboratory of Magnetic Resonance and Atomic Molecular Physics, Wuhan Institute of Physics and Mathematics of the Chinese Academy of Sciences, Wuhan, Hubei Province 430071, China; 2 Division of Computational Bioscience, Center for Information Technology, National Institutes of Health, Building 12A, Bethesda, MD 20892, United States of America; National Institute for Medical Research, Medical Research Council, London, UNITED KINGDOM

## Abstract

More than 40% of the RNA structures have been determined using nuclear magnetic resonance (NMR) technique. NMR mainly provides local structural information of protons and works most effectively on relatively small biomacromolecules. Hence structural characterization of large RNAs can be difficult for NMR alone. Electron microscopy (EM) provides global shape information of macromolecules at nanometer resolution, which should be complementary to NMR for RNA structure determination. Here we developed a new energy term in Xplor-NIH against the density map obtained by EM. We conjointly used NMR and map restraints for the structure refinement of three RNA systems — U2/U6 small-nuclear RNA, genome-packing motif (Ψ^CD^)_2_ from Moloney murine leukemia virus, and ribosome-binding element from turnip crinkle virus. In all three systems, we showed that the incorporation of a map restraint, either experimental or generated from known PDB structure, greatly improves structural precision and accuracy. Importantly, our method does not rely on an initial model assembled from RNA duplexes, and allows full torsional freedom for each nucleotide in the torsion angle simulated annealing refinement. As increasing number of macromolecules can be characterized by both NMR and EM, the marriage between the two techniques would enable better characterization of RNA three-dimensional structures.

## Introduction

Non-coding RNAs are essential in many aspects of life [1–6]. The secondary structures of RNA molecules can be quite accurately predicted [7,8]. However, it remains difficult to determine the three-dimensional structure of large RNAs experimentally, let alone *in silico* prediction. RNA is intrinsically dynamic [9,10], and it can be difficult to crystalize for structural studies using X-ray crystallography. Nuclear magnetic resonance (NMR), on the other hand, determines macromolecule structures in solution, and can be uniquely suited to characterize RNA structures. Indeed, to this date, RNA structures determined by NMR make up >40% of the total RNA structures deposited at the nucleic acid database (NDB). In contrast, protein structures determined by NMR make up only less than 10% of the total protein structures in the protein data bank (PDB).

An RNA molecule is a polymer of four types of nucleotides, compared to 20 amino acids in a protein. Owing to the low chemical complexity in RNA primary sequence, the chemical shift dispersion is small and the NMR spectra are often poorly resolved. Moreover, an RNA molecule has a lower density of protons than a protein of the same molecular weight, hence fewer distance restraints per nucleotide can be obtained from the measurement of proton-proton nuclear Overhauser effect (NOE) [11]. The NOE distance restraint is the classic yardstick in NMR, but is semi-quantitative at best and local by nature, involving protons that are separated by less than 6 Å. Therefore, if solely based on the NOE distant restraints, cumulative errors can build up when determining the structures of large RNAs [12]. Together, RNAs that have been structurally characterized by NMR averages only about 24 nucleotides in length [13], corresponding to a molecular weight of ~8 kDa. As such, to better determine RNA structures and to characterize larger RNAs using NMR, long range and global experimental restraints are needed.

Besides NOE distance restraints, other types of restraints have been incorporated into the RNA structure determination. Residual dipolar coupling (RDC), a type of NMR experiment, provides bond orientation information—often measured for imino groups of RNA—relative to an alignment tensor [12,14]. Small angle X-ray scattering (SAXS), on the other hand, provides the averaged shape information of a biomacromolecule in solution, and has been used in conjunction with NMR restraints [15]. Wang and co-workers developed a top-down approach called G2G for refining the RNA structure. They were able to resolve the degeneracy inherent to RDC based on SAXS global shape information, and determined the relative angles between duplexes in an RNA molecule [16]. In the subsequent refinement, the authors fixed the orientations of RNA duplexes, and only gave full torsion freedom to the linker nucleotides [16–18]. Thus, it is particularly important that the input RDC and SAXS data are of high quality and the RNA starting structure has been correctly assembled. Other issues can be associated with SAXS measurement for RNA. For example, RNA is prone to aggregation, especially at high concentration required for SAXS data collection [2], which can obscure the native RNA structure. Even in the absence of aggregation, variable ligand occupancy, different oligomerization states, and multiple conformations of the RNA may complicate the scattering profile.

Electron microscopy (EM) has become an important technique in structural biology. EM affords global shape information of a macromolecule at nanometer resolution, which should complement the local structural information provided by NMR. Indeed, when the structures of each component are known, large macromolecular assemblies can be modeled by integrating various experimental inputs including both NMR and EM [19]. However, NMR works most comfortably on biomacromolecules of less than 50 kDa in molecular weight, especially when *de novo* determining the structure of a single-chain polypeptide or nucleic acid. On the other hand, EM micrographs suffer from low image contrast, especially for relatively small biomacromolecules. Recent development in cryogenic electron microscopy (cyro-EM), cryogenic electron tomography (cyro-ET), and single-particle reconstruction, however, has permitted more routine visualization of biomacromolecules with molecular weight below 200 kDa [20]. In addition, since an RNA molecule contains more heavy atoms including ^31^P than a protein of the same molecular weight (at the expense of lower proton density), an RNA molecule diffracts more electrons than a protein does. All told, the stage is set to marry EM with solution NMR for the RNA structure refinement.

## Methods

To incorporate the EM data, we developed a new density map potential, and we implemented this term in Xplor-NIH molecular structure determination package [21], version 2.36 and later. The map potential term assesses the cross-correlation between the input map and the atomic probability [22] calculated from the macromolecule coordinates, which is defined as,

$$C=\frac{1}{N}\overset{n}{\underset{i=1}{\sum }}\frac{\left(m_{i}^{obs}-\bar{m^{obs}}\right)\left(m_{i}^{calc}-\bar{m^{calc}}\right)}{\sigma^{obs}\sigma^{calc}}$$

in which, $m_{i}^{obs}$ is a value in the input map, $m_{i}^{calc}$ is the corresponding calculated value, $\bar{m^{obs}}$ and *σ* ^*obs*^ are the average and standard deviation of the input map, and $\bar{m^{calc}}$ and *σ* ^*calc*^ are the average and standard deviation of the back-calculated values from the RNA structure. When optimizing C, the energy associated is *k*c(1-C), in which *k*c is the force constant scale. To invoke the map potential term in an Xplor-NIH python script, the following snippet is incorporated.

from atomDensity import AtomDensity, DensityGrid

Dmap = DensityGrid()

Dmap.readCCP4('map1.ccp4',verbose = True)

from probDistPotTools import create_probDistPot

prob = create_probDistPot("prob",Dmap,

"not pseudo and not name H*",

potType = "cross_correlation",)

potList.append(prob)

prob.setScale(10)

The density map potential term currently only reads maps in CCP4 format (the file name here is map1.ccp4), which can be generated experimentally (see below) or downloaded from EM DataBank (EMDB). For the two examples presented in this study, U2/U6 small-nuclear RNA [18] and ribosome binding structural element from turnip crinkle virus [17], we generated the corresponding maps using the program pdb2vol in the Situs package [23] from the known PDB structures, with 2 Å^3^ voxel size and Gaussian smooth kernel at various resolutions.

In a typical Xplor-NIH run, the restraint table includes both covalent geometrical (bond, angle, and impropers) and experimental restraints including NOE distance and RDC orientation restraints. Based on RNA secondary structure, hydrogen bonding, base planarity, and dihedral angle restraints [24] were employed to enforce regular A-form helices. Knowledge-based quadratic torsion-angle database, RNA-specific base-base positioning potentials [25], and backbone P-P distance restraints [13] were also imposed. During simulated annealing refinement, the RNA molecule was first heated to 3000 K and was equilibrated for 50 ps, with full torsion-angle freedom given to every nucleotide. The bath temperature gradually cooled from 3000 K to 25 K, with 200 steps; at each temperature, torsion-angle molecular dynamics ran for 0.5 ps. The calculation was repeated 128 times; from the 128 structures calculated, 20 structure models were selected for their lowest energy and smallest RMS deviations from each other. All structure figures were rendered using UCSF Chimera [26].

## Results

### Refinement of U2/U6 small-nuclear RNA structure

**Fig 1:**
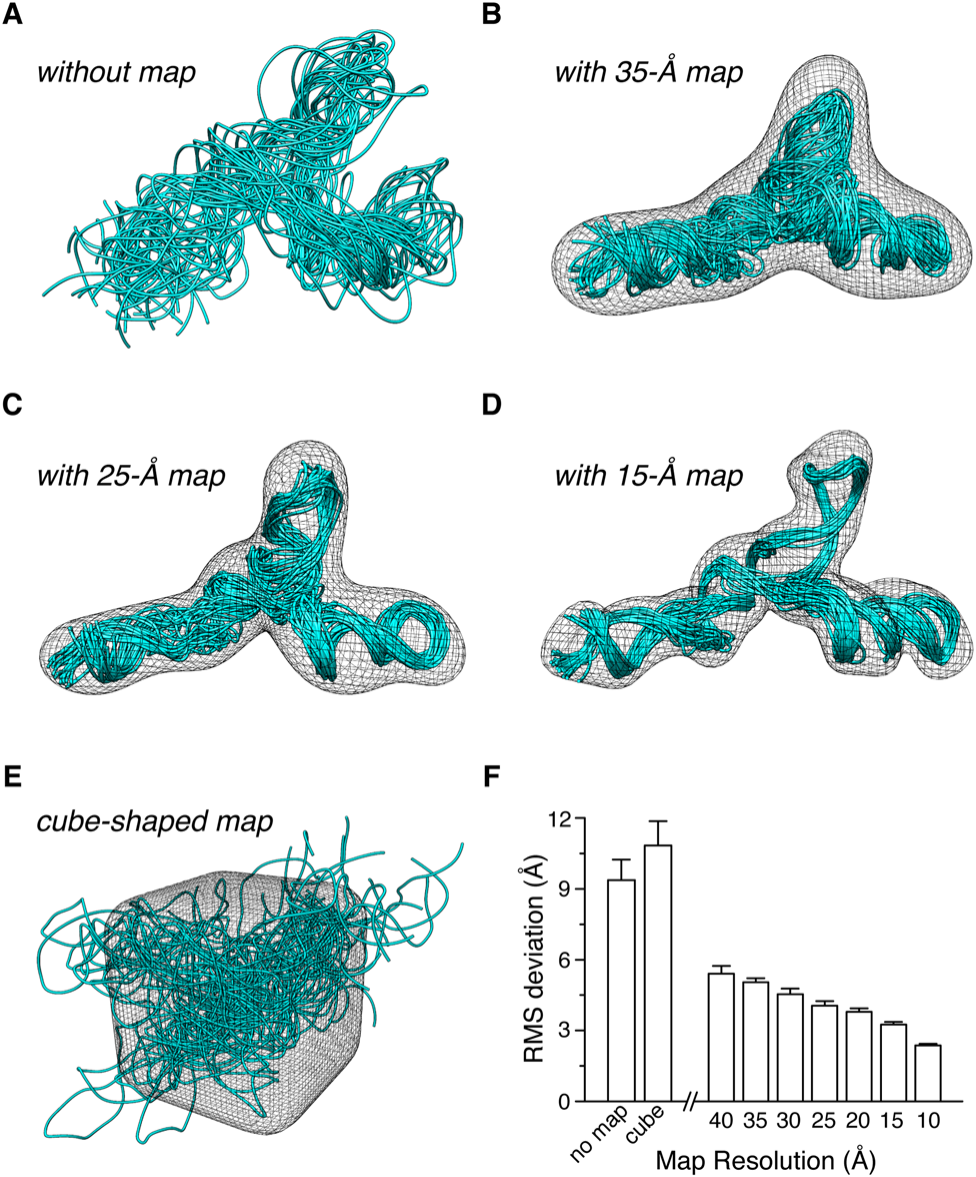
Structure refinement of the U2/U6 small-nuclear RNA. (A) Superposition of 20 structures calculated by refining against NMR, secondary structure, and knowledge-based restraints. The RMS deviation is 9.87±0.87 Å. (B-D) Upon the incorporation of the maps at 35 Å, 25 Å, and 15 Å resolutions, the RMS deviation for the 20-model bundle can be improved to 5.05±0.17 Å, 4.05±0.19 Å, and 3.25 ± 0.11 Å, respectively. (E) Upon the incorporation of an artificial, cube-shaped density map restraint, the structural convergence deteriorates to 10.84±1.02 Å. (F) Histogram showing the relationship between RMS deviation value and the resolution of the input map.

We first examined the 111-nucleotide U2/U6 small-nuclear RNA (snRNA) involved in pre-RNA splicing. U2/U6 comprises a three-way junction arranged in a Y-shape, and its three-dimensional structure has been determined by refining against both NMR and SAXS data. Unfortunately, only the NMR restraints are available from the PDB, whereas the associated SAXS data have not been deposited by the authors [18]. We performed the structural refinement against available NMR restraints using Xplor-NIH [21]. Despite all the experimental and knowledge-based restraints, the afforded snRNA structure is poorly converged, with a root-mean-square (RMS) deviation of 9.37±0.87 Å for all heavy atoms (Fig. 1A).

We generated the density map using the program pdb2vol in the Situs package [23] at different theoretical resolutions, based on the structure of U2/U6 snRNA (PDB accession code 2LKR, first model). Upon incorporation of the density map restraint into the refinement, the structural convergence of U2/U6 snRNA can be greatly improved—even using a density map with a theoretical resolution of only 40 Å, the RMS deviation can be improved to 5.41±0.33 Å; with a 35-Å map, the RMS deviation can be lowered to 5.05±0.17 Å (Fig. 1B). As the resolution of the input map improves, the precision of the afforded U2/U6 snRNA structure improves accordingly (Fig. 1F). Upon the incorporation of a 15-Å resolution map, in which the major groove is already visible, the precision of the coordinates can be improved to 3.25±0.11 Å in RMS deviation (Fig. 1D). With the input of a 10-Å map restraint, the RMS deviation for a 20-model ensemble calculated is only 2.37±0.07Å, and RMS difference from the reference structure is about the same—3.25±0.16 Å from the first model of 2LKR PDB structure. As a negative control, we generated a cube-shaped density map. Incorporation of this artificial restraint significantly deteriorates the structural precision and accuracy (Fig. 1E), with 10.84±1.02 Å in RMS deviation for the 20-model ensemble and 19.21±0.96 Å in RMS difference from the reference structure. Taken together, even with the input of a very low-resolution map, as long as it is correctly identified corresponding to the RNA molecule, the structure can be refined to better precision and accuracy.

### Structural refinement of retroviral genome packing motif

The retroviral genome-packing motif (Ψ^CD^)_2_ from Moloney murine leukemia virus (MMLV) is a dimer with 66 nucleotides in each RNA chain, with a total molecular weight of 42.8 kDa. The (Ψ^CD^)_2_ has been characterized with cryo-ET—the density map was derived with sub-volume averaging from only 47 particles [2], and appears to have a resolution of no better than 30 Å. In the previous study, the RNA structure was obtained by refining against the extensive NMR restraints (PDB accession code 2L1F), and was only qualitatively compared to the cryo-ET density map [2].

**Fig 2:**
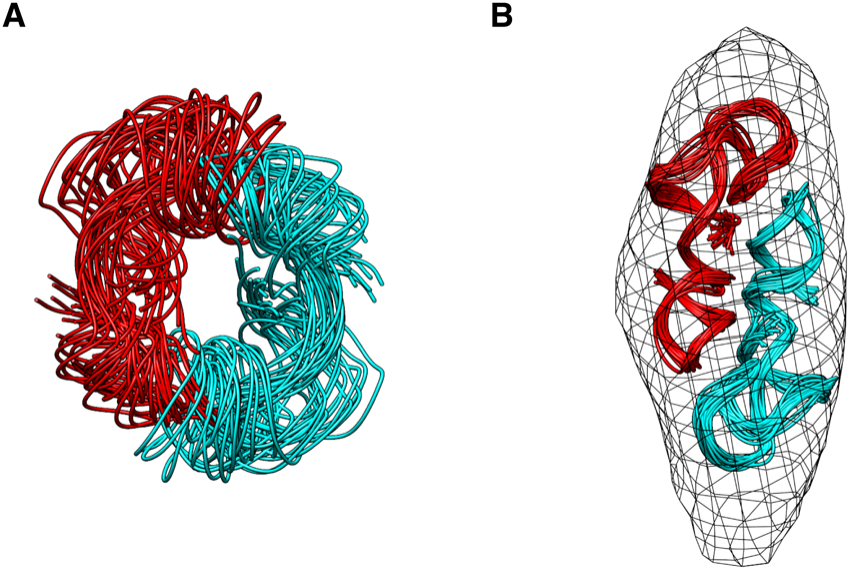
Structure refinement the genome-packing motif (Ψ^CD^)_2_ from Moloney murine leukemia virus. Comparison of the (Ψ^CD^)_2_ RNA dimer structure calculated (A) without or (B) with cryo-ET density map restraint. The two RNA subunits in (Ψ^CD^)_2_ homodimer are colored magenta and cyan, respectively. 20 structural models were superimposed, affording RMS deviations of 3.37±0.45 Å and 1.67±0.33 Å, respectively. The cryo-ET map (EMDB accession code 1806) is shown as meshes in (B) with a threshold of 6.

We repeated the RNA structure calculation using Xplor-NIH [21] against covalent, knowledge-based, NOE, hydrogen bond, base planarity, and dihedral angle restraints. In addition, non-crystallographic symmetry was applied to enforce (Ψ^CD^)_2_ dimeric arrangement. Refined without map restraint, the afforded structures converge to an RMS deviation of 3.37±0.45 Å for all heavy atoms in both chains of the (Ψ^CD^)_2_ (Fig. 2A). With the incorporation of the map restraint, the precision of the (Ψ^CD^)_2_ is greatly improved, to an RMS deviation of 1.67±0.33 Å (Fig. 2B). The (Ψ^CD^)_2_ is a homodimer and contains fewer secondary structure elements than U2/U6 snRNA, which can explain why better structural convergence is achieved for (Ψ^CD^)_2_. Importantly, our calculation demonstrated that the RNA structure could be refined to better precision, with the incorporation of a cryo-ET map of a rather poor resolution.

### Structural refinement of ribosome-binding element

**Fig 3:**
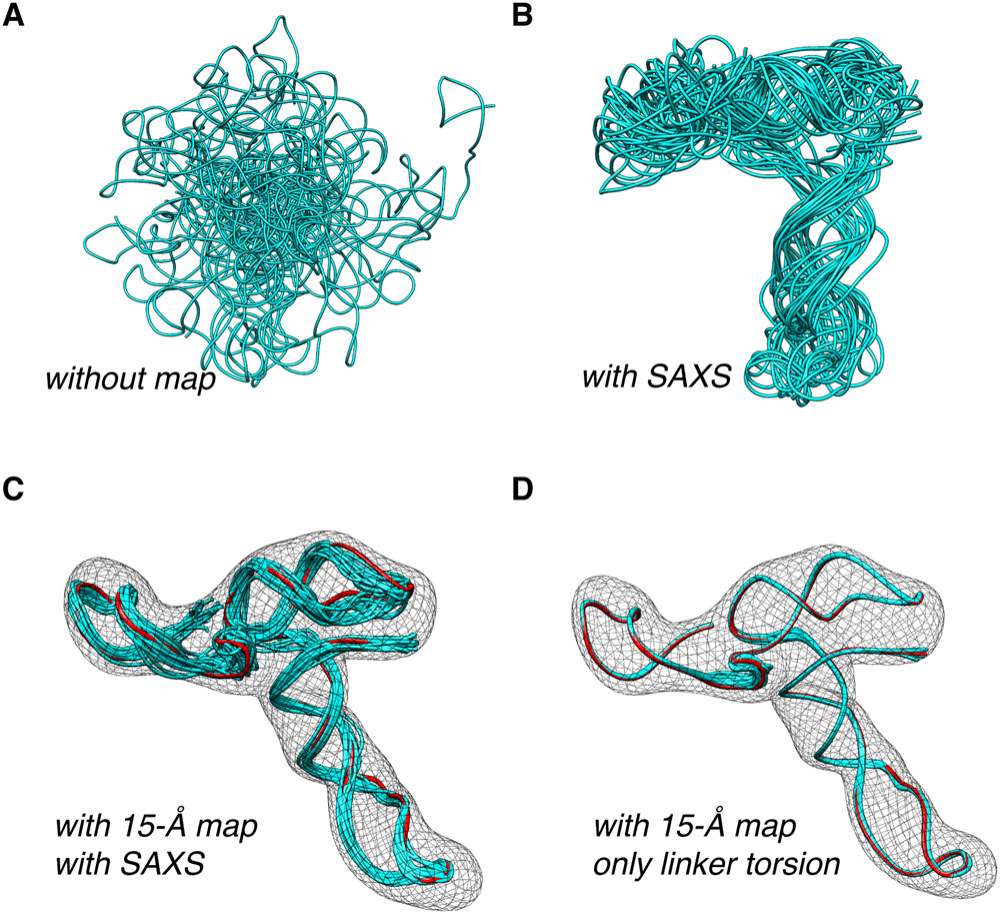
Structure refinement of ribosome-binding element from turnip crinkle virus genome. (A) Superposition of 20 structures, obtained by refining against NMR, secondary structure, and knowledge-based restraints. The RMS deviation for the bundle is 13.24±0.56 Å. (B) When incorporating of SAXS and P-P envelope distance restraints, the structural convergence is improved to 8.20±0.44 Å. (C) With the incorporation of additional map restraints, the RMS deviation is further lowed to 2.35±0.05 Å, for the 20-structure bundle. (D) By allowing only translation movement for the RNA duplexes but not the rotational movement, refined against both NMR and map restraints, the convergence of the calculated structures can be improved to 0.76±0.03 Å. The structure for generating the density map (the first model of PDB structure 2KRL) is colored red. The RMS difference between the structure calculated and the reference structure is 2.65±0.27 Å and 0.94±0.04 Å for (C) and (D), respectively.

We further tested our method on a 102-nucleotide ribosome-binding element (RBE) from the 3’-untranslated region of turnip crinkle virus genome. Shaped like a tRNA, the solution structure of RBE was previously characterized with joint refinement against NMR and SAXS data, to an RMS deviation of 1.4±0.2 Å for a 10-model bundle [17]. We repeated the calculation, first with only NMR, secondary-structure and knowledge-based restraints. The structural convergence is poor, with RMS deviation of 13.24±0.56 Å (Fig. 3A). We then incorporated the SAXS restraint [17] and the P—P distance restraints derived from SAXS global shape information [17,18], and we were able to refine the RBE structure to an RMS deviation of 8.20±0.44 Å for a 20-model bundle (Fig. 3B). Based on the published structure of RBE (PDB accession code 2KRL, the first model), we generated a 15-Å resolution map using Situs [23]. Upon incorporating the additional map restraint, the RBE structure can be refined to 2.35±0.05 Å in RMS deviation (Fig. 3C). Importantly, the RMS difference between our structure and the reference PDB structure is only 2.65±0.27 Å.

But why is the RMS deviation for the structure we calculated with SAXS and NMR restraints (8.20±0.44 Å) much larger than the value previously reported (1.4±0.2 Å)? In the previously study [17], the authors used the G2G approach [16]—they analyzed NMR RDC and SAXS data, and determined the relative orientations between the four RNA helices of RBE. They then assembled the RNA duplexes [16,27] to obtain an initial RBE structure. In the subsequent refinement, the RNA duplexes can only translate but not rotate, and full torsional freedom was only given to the linker between RNA duplexes. Therefore, it was particularly important to have a good starting structure of the RNA molecule. In our approach, however, we do not construct a model *a priori* and we give full torsional freedom to each nucleotide in the RNA. For a fairer comparison, we fixed the relative angles between the duplexes in RBE when incorporating additional map restraint but in the absence of the SAXS restraint. We were able to refine the RBE structure to an RMS deviation of 0.76±0.03 Å for a 20-structure bundle (Fig. 3D) and the RMS difference from the reference structure of 0.94±0.04 Å. Taken together, with the introduction of the new density map potential in Xplor-NIH, the RNA structure can be refined to better precision and accuracy.

## Discussion

In this study, we developed a new potential term in Xplor-NIH [21], and we incorporated the map restraint for the structure refinement of non-coding RNAs. By using both NMR and map restraints, we showed that the RNA structure can be refined to higher precision and accuracy, even with a map at a resolution as low as 40 Å. With the recent development in cryo-EM and cryo-ET, biomacromolecules smaller than 200 kDa can be more routinely characterized. This can be particularly the case for RNA, as RNA contains more heavy atoms than protein [2,28]. We also realized that the density map is not limited to cryo-EM or cryo-ET, 3D reconstruction of maps from negative staining EM [29] can be equally helpful for the RNA structure refinement.

The fact that NMR spectroscopy is most effective for biomacromolecules less than 50 kDa has been a major bottleneck for this technique: as the system gets larger, NMR peaks become broader and more peaks can overlap. Notwithstanding, technical development in recent years is pushing the limit on the size of macromolecules that NMR can characterize [30]. Though the NMR spectrum for RNA is less well dispersed than that of a protein, an RNA molecule can be readily isotopically labeled at selected nucleotides or segments [31], and different segments of an RNA can be inspected using a divide-and-conquer approach. Together, the “large” RNAs that can be characterized by NMR are beginning to overlap with those “small” RNAs that can be characterized by EM. We envision that the marriage between NMR and EM should allow better depiction of RNA structures.
